# The Effect of Mindfulness on the Promotion of Graduate Students’ Scientific Research Creativity: The Chain Mediating Role of Flow Experience and Creative Self-Efficacy

**DOI:** 10.3390/jintelligence12030024

**Published:** 2024-02-21

**Authors:** Hao Yao, Yabing Fan, Shifei Duan

**Affiliations:** 1 Institute of Higher Education, Tongji University, Shanghai 200092, China; 2 College of Education, Zhejiang University, Hangzhou 310058, China

**Keywords:** scientific research creativity, mindfulness, flow experience, creative self-efficacy, mediating effect

## Abstract

Creativity is a universal core higher-order cognitive ability in the 21st century, which reflects a country’s core competitiveness and soft power. Mindfulness, as an important concept in positive psychology, has also received attention for its potential effect on research creativity. Using structural equation modeling and bootstrap methods, this study investigated the relationship between mindfulness and research creativity among 1210 Chinese graduate students. Additionally, we explored the mediating effect of flow experience and creative self-efficacy in this relationship. The research results showed that mindfulness had a direct positive effect on graduate students’ scientific research creativity. The mediating effect of flow experience and creative self-efficacy, as well as the chain mediating effect, were established, with the mediating ratio being 13.1% and 30.0%, respectively, and the indirect effect of chain mediating accounting for 34.1%. Interestingly, the effect mechanism at the gender level was various, with the mediating effect of mindfulness on scientific research creativity being higher among male graduate students. The results of this study revealed the mechanism of mindfulness on graduate students’ scientific research creativity, offering valuable insights for enhancing their creative capabilities in the realm of scientific research.

## 1. Introduction

With the fourth industrial revolution represented by artificial intelligence and the rapid development of the knowledge economy, creativity has become an indispensable ability for high-quality human resources (Jauk et al. 2015). Creativity is a core higher-order cognitive ability positioned as a universal core competence and a key thinking quality in the 21st century, embodying a country’s core competitiveness and soft power and, also, acting as a key factor affecting a country’s technological competitiveness (Henriksen et al. 2016; Henriksen et al. 2018). The World Economic Forum recognizes creativity as one of the three essential skills that citizens must have to cope with the fourth industrial revolution (the other two are the ability to solve complex problems and the ability to think critically) (Frick and Brodin 2020). In response to the demands of the evolving future society, the Programme for International Student Assessment (PISA) launched by the Organization for International Economic Cooperation and Development (OECD) added creative thinking assessment items for students from all participating countries in the new PISA assessment in 2022 (OECD 2019). This move aims to emphasize the significance of nurturing students’ critical and creative abilities.

In the era of the Anthropocene, beginning with the global effect of human activities on climate and ecosystems in the late 18th century, the manifestations of the Anthropocene are now most evident in issues such as climate change, pollution, the global pandemic, and the social crises that they cause. As a distinctly human attribute (Henriksen et al. 2022b), creativity holds the potential to address these challenges effectively. In the field of education, creativity management and scientific innovation offer some promising solutions to the complex problems in the Anthropocene (O’Doherty 2020). Bruno Latour (2018) describes the Earth as an unpredictable “terrestrial attractor” while society faces too much uncertainty. This new geological era requires the development of creative scientific leaders and the adoption of innovative educational methods and strategies to enhance students’ scientific creativity. The need for creativity in the Anthropocene has also been raised by a number of researchers (Cheng 2019; Sandri 2013). Therefore, in order to explore new directions for contributing to sustainable development in the world and focus on the potential of research creativity to help us address current challenges and anticipate future ones, there is a need to further explore and examine how postgraduate research creativity can be enhanced and what factors are considered critical.

“Scientific research creativity” is a concentrated expression of “creativity” in the context of academic research, which is widely believed to be capable of generating novel and valuable ideas. It specifically refers to the comprehensive literacy and ability to systematically apply theoretical knowledge and creatively solve problems, emphasizing the novelty and applicability of research issues, tools, processes, and perspectives (McWilliam 2009; Liu et al. 2020). The characteristics of scientific research creativity include cognitive, psychological, and sociocultural aspects (Glăveanu 2011). The cognitive and psychological aspects refer to the ability to generate innovative ideas, advocating that scientific research be viewed as creative work and deep thinking. Moreover, creativity is considered to have sociocultural attributes because the output of scientific research creativity relies on the support of social resources and those values need to be recognized in the context of a sociocultural environment (Glăveanu 2018).

Universities are the main source of knowledge creation, especially for graduate education, which represents the highest level of talent cultivation within the university system. Students, including both master’s and doctoral students, are the main body of knowledge creation and innovation output. Consequently, universities around the world have also expanded their enrollment rates in various fields (Shin et al. 2018). The quality of graduate student training, especially the level of scientific research creativity, largely determines the quantity and quality of innovative talents. Therefore, enhancing graduate students’ scientific research creativity is considered an important indicator for evaluating the quality of higher education (Chan and Ngok 2011). Universities are also increasingly emphasizing the creative use of new technologies and methods in course instruction to promote creative teaching and learning (Livingston 2010). However, scientific research creativity, as an important feature of graduate student performance or as a factor in the development of higher education institutions, has received little academic attention (Frick and Brodin 2020). Some studies have pointed out that the current prospects for the development of graduate students’ creativity do not seem optimistic because universities do not always promote graduate students’ research creativity (Brodin 2016, 2018).

Many studies exploring the influencing factors of research creativity are mostly from the perspectives of individuals, mentors, and situations (Chen and Cheng 2023; Liu et al. 2020), including positive psychological attributes of individual factors, such as mindfulness meditation, curiosity, risk tolerance, motivation, self-efficacy, and willingness to overcome failure (Heinze et al. 2009; Brodin 2016; Fan et al. 2019). Tutor factors include, for example, independent support from mentors (Jianlin et al. 2014), academic leadership (Meng and Zhao 2018), and mentor feedback (Chen et al. 2021). Scenario factors, including team size and department effects (Fan et al. 2019), are also considered in the analysis of research creativity.

Mindfulness is an important concept in positive psychology, which has recently received widespread attention in academic and popular discourse (King and Badham 2020). As a positive psychological attribute of individuals, mindfulness has also gained attention for its effect on scientific research creativity; similarly, some studies have shown that mindfulness plays a positive role in stimulating and promoting creativity (Yousaf and Taylor 2022; Schutte and Malouff 2020). Issues such as increasing employment pressure, information overload, and research anxiety are commonly considered barriers to creativity; however, mindfulness can effectively address these barriers (Fisher 2006). Some studies have suggested that mindfulness could alleviate anxiety symptoms and reduce the fear of being judged by others by improving stress regulation (Shapiro et al. 2006; Carson and Langer 2006; Michel et al. 2021). Therefore, the positive effect of mindfulness has been fully reflected in university education (Frick and Brodin 2020). Although mindfulness does not necessarily provide practical solutions to the difficulties faced by individuals, it has a good effect on solving emotional and cognitive problems caused by stress or psychological trauma.

Indeed, while previous research has primarily focused on enterprise employees, it is essential to recognize that graduate students play a vital role as key talents for social innovation and entrepreneurship. As such, their research creativity deserves attention. However, there is a lack of research on the relationship between the mindfulness of graduate students and research creativity (Gip et al. 2022; Ngo et al. 2020; Byrne and Thatchenkery 2019). In addition, in terms of the mediating mechanism between mindfulness and scientific research creativity, some studies have used self-efficacy or flow experience alone as the mediating variable between mindfulness and creativity, which fully demonstrates that there is a certain link between mindfulness, creativity, self-efficacy, and flow experience (Mendonça et al. 2018; Chen et al. 2022). There is no doubt that the research on the relationship and effect mechanism between mindfulness and graduate research creativity is worth exploring by scholars (Henriksen et al. 2020).

Therefore, this study constructs a chain mediating model to explore the relationship between mindfulness and graduate students’ scientific research creativity and its mechanism so as to examine the chain mediated role of flow experience and creative self-efficacy. This not only broadens the application field of mindfulness but also provides a new perspective on how to improve graduate students’ scientific research creativity.

### 1.1. Mindfulness and Scientific Research Creativity

Mindfulness, with its origins in Eastern Buddhism, is rooted in the concept of lucidity and concentration of mind (Sharf 2014). In the classical Buddhist context, mindfulness emphasizes attention and awareness of the present state (Quaglia et al. 2015). It refers to an individual’s conscious and non-judgmental attention to internal and external stimuli at the present moment (Kabat-Zinn 2003).

In the process of enhancing creativity, the differences between Chinese and Western cultures have also received attention. In Eastern cultures, mindfulness is seen as the key to enhancing creativity while Western cultures believe that creativity is primarily achieved through social interaction (Shao et al. 2019). Some studies have found that mindfulness can enhance creativity (Ostafin and Kassman 2012; Henriksen et al. 2020). Mindfulness requires individuals to perceive and accept external stimuli with a free and calm mindset to promote divergent thinking and enhance creativity (Henriksen et al. 2020). Richard et al. (2017) also found that under the influence of mindfulness, individuals are more likely to develop an “epiphany” state during problem thinking, achieving higher levels of insight, which is an important component of creative thinking.

Mindfulness also enhances creativity by promoting individual psychological emotions, as negative emotions, such as distraction and anxiety, can harm attention and thinking abilities (O’Donnell 2015). Mindfulness can mitigate these negative emotions and control attention by reducing the attention paid to distracting information (Wadlinger and Isaacowitz 2011). Task-related intelligence and creativity can be enhanced by providing a high level of concentration (Vogus and Sutcliffe 2012). Therefore, we developed the following hypothesis:

**Hypothesis** **1.** *Mindfulness can positively predict the scientific research creativity of graduate students.*

### 1.2. Mediation of Flow Experience

Mindfulness is the premise and foundation of flow experience (Csikszentmihalyi 1997; Kaufman et al. 2009), which was first proposed by Csikszentmihalyi in the field of positive psychology, referring to the optimal emotional experience that occurs when an individual focuses on solving a problem or participating in an activity, driven by intrinsic motivation and perseverance (Csikszentmihalyi 1975; Kimiecik and Stein 1992). When an individual is in flow experience, their consciousness is all concentrated in a narrow channel, automatically filtering out information unrelated to the task. If you want to propose innovative ideas in the scientific research field, you need to analyze objective things rationally, which combines mindfulness with flow experience (Briegel-Jones et al. 2013). Although mindfulness involves maintaining reflective awareness while flow experience is about losing consciousness, that is, absorption, scholars have proven that mindfulness may interfere with the absorption facet of flow but not with other aspects of flow (Sheldon et al. 2015). Marty-Dugas et al. (2021) proposed that mindfulness may be positively associated with flow as both mindfulness and flow focus on engagement with the present moment. Some research has supported the proposition that greater mindfulness is associated with higher levels of flow (Schutte and Malouff 2023).

Flow experience can also have an effect on graduate students’ scientific research creativity. As flow experience is a high degree of internal motivation and creativity is generated by an individual’s internal drive, when graduate students are experiencing flow, they are more likely to generate scientific research creativity. Some scholars believe that individuals are most creative when they are at the peak of their flow experience (Heerden 2010). When attention is highly focused on the current task, consciousness and spirit are highly concentrated then flow experiences occur, as well as insight becoming more likely, thereby stimulating and promoting creativity (Yang et al. 2019). Therefore, mindfulness not only has a direct positive effect on creativity but also can have an indirect positive effect on creativity through flow experience (Chen et al. 2022). Accordingly, we developed the following hypothesis:

**Hypothesis** **2.** *Flow experience plays a mediating role between mindfulness and scientific research creativity.*

### 1.3. Mediation of Creative Self-Efficacy

Self-efficacy, first proposed by Bandura in 1977, refers to an individual’s subjective judgment in their capacity to accomplish a specific activity, reflecting their confidence in achieving behavioral goals in a particular domain. (Bandura 1977). Creative self-efficacy is the expression of self-efficacy in the creative field, which refers to an individual’s belief in their ability to produce creative results (Tierney and Farmer 2002).

Mindfulness can focus on the present and maintain a receptive attitude towards current events. On the basis of having a wealth of information and diverse technical skills, individuals believe that they can generate new ideas and creatively solve problems, thereby helping to improve their sense of creative self-efficacy (Mendonça et al. 2018). The most obvious feature of mindfulness is the function of self-regulation. The improvement of the mindfulness ability of graduate students can help optimize their self-regulation ability (Glomb et al. 2011), so as to keep a better emotional state when engaged in scientific research, with a higher sense of creative self-efficacy.

According to social cognitive theory, individual self-efficacy is a key factor that determines individual decision-making and behavior (Bandura 1977). Creative self-efficacy is also considered an influential mechanism for enhancing creativity (Wang et al. 2018). Some scholars pointed out that the realization of creative achievements requires flexible cognitive styles and a lot of detailed work (Amabile 1993). Individuals with a high sense of creative self-efficacy have the advantage of widely collecting information, breaking thinking patterns, and believing that they can solve problems, thus investing more energy in scientific research (Deci and Ryan 1987; Zhang et al. 2022). Some scholars also support the positive relationship between creative self-efficacy and creativity based on empirical results (Tierney and Farmer 2004). Therefore, mindfulness can further enhance creativity through a sense of creative self-efficacy (Mendonça et al. 2018). Therefore, we developed the following hypothesis:

**Hypothesis** **3.** *Creative self-efficacy plays a mediating role between mindfulness and scientific research creativity.*

### 1.4. The Chain Mediating Effect of Flow Experience and Creative Self-Efficacy

There may be a positive relationship between flow experience and creative self-efficacy. Flow experience positively predicts self-efficacy. The higher an individual’s flow experience level, the more likely they are to set clear goals (Mao et al. 2020) and the stronger the sense of immersion and pleasure they experience when completing tasks, resulting in a higher sense of creative self-efficacy (Csikszentmihalyi 1975). In addition, flow experience can also have a positive effect on the independence and freedom of individual activities, which enhances creative self-efficacy to some extent (Yeh et al. 2019). Individuals with higher scores of creative self-efficacy tend to be more creative and achieve higher innovative outputs (Gong et al. 2009; Wang et al. 2018). Therefore, we developed the following hypothesis:

**Hypothesis** **4.** *Flow experience and creative self-efficacy play a chain mediating role between mindfulness and scientific research creativity.*

**Figure 1:**
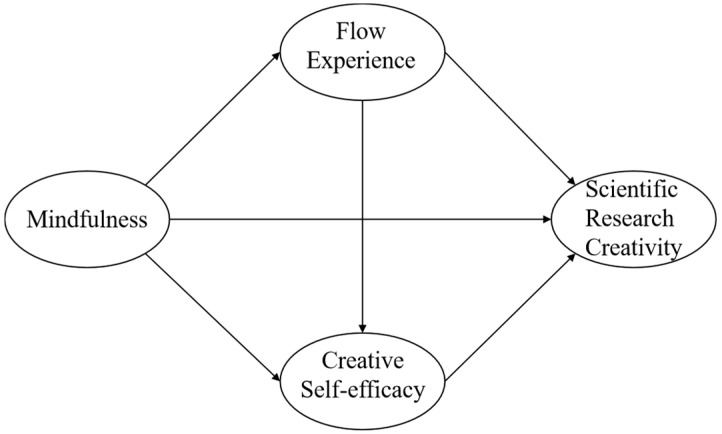
The hypothesized model.

According to the above literature review, this study explores the relationship between mindfulness and scientific research creativity among Chinese graduate students. In addition, we hypothesize that the relationship is influenced by a chain mediating role between flow experience and creative self-efficacy (Figure 1).

## 2. Materials and Methods

### 2.1. Participants

This study mainly collected data from postgraduates studying in universities located in three provinces in eastern China, including Jiangsu, Zhejiang, and Shanghai. Considering the stratification of the Chinese higher education system, a random sampling method based on the institutional level was used to conduct a questionnaire survey. We contacted the university’s information technology department and distributed the questionnaire to students after obtaining official approval. We informed participants of the background and purpose of this survey. Creativity is a universal core higher-order cognitive ability that has become indispensable for quality human resources. Exploring the relationship between mindfulness and graduate students’ scientific research creativity and the effect of influencing mechanisms is the main thrust of this investigation. The link to the online survey was sent to graduate students via academic email and WeChat notifications. When questionnaires were distributed, there was a consultation with graduate students to fully inform all participants of the reasons for this study and how the research data would be used. Research Statement: (1) This survey is not about personal illnesses, habits, and other private issues but only about personal aspects of mindfulness and creativity; (2) This survey is anonymous and no personal information will be published; (3) The raw data of this survey will not be used publicly, they will only be used by our research team. And only after seeking the consent of the graduate students to tick the ‘I voluntarily participate in being surveyed’ option, can they fill in the questionnaire further.

A total of 1210 valid questionnaires were collected out of 1250 distributed, with an effective questionnaire rate of 96.8%. The demographic statistics of this study’s participants included 42.9% (n = 519) men and 57.1% (n = 691) women in terms of gender. Regarding academic levels, 61.7% (n = 747) of the respondents were master’s students and 38.3% (n = 463) were doctoral students. In terms of disciplines, postgraduate students in humanities (literature, history, and philosophy) accounted for 22.2% (n = 269); social sciences (economics, management, law, education, and society) accounted for 45.9% (n = 555); and engineering science, agriculture, and medicine accounted for 21.9% (n = 386).

### 2.2. Materials

#### 2.2.1. Special Quality Table of Mindfulness

Using the Cognitive and Affective Mindfulness Scale-Revised (CAMS-R) developed by Feldman et al. in 2007, the four components of mindfulness, namely, attention, consciousness, current focus, and acceptance were measured (Feldman et al. 2007). These four components are emphasized as the core theme of mindfulness in several definitions (Bishop et al. 2004). Participants responded to 12 items on a 5-point scale ranging from 1 (very little/no) to 5 (almost always) and then we calculated the overall mindfulness score. Sample items are “It is easy for me to concentrate on what I am doing” and “I am able to pay close attention to one thing for a long period of time”. CAMS—R has been widely used in the field of graduate students, as exemplified by Sinha, who had used the method to measure the mindfulness traits of management graduate students (Sinha et al. 2021).

#### 2.2.2. Scientific Research Creativity of Graduate Students

The scientific research creativity dimension for graduate students used the Six-Item Scale for Student Creativity developed by Madjar et al. (2011). The key indicators are creative thinking, solutions, and teamwork. Participants were asked to rate these items on a 5-point scale, ranging from 1 (very disagree) to 5 (very agree) (Madjar et al. 2011). The scale has been translated into Chinese and applied to measure graduate students’ scientific research creativity (Tang and Ding 2014). Sample items are “I can formulate original and relevant research questions” and “I demonstrate originality in teamwork”.

#### 2.2.3. Flow Experience

The Short Dispositional Flow Scale 2 (SDFS-2) was used for the flow experience dimension of graduate students. The scale, developed by Jackson et al. (2008), includes eight items: challenging awareness, action awareness integration, clear goals, clear feedback, task focus, enhanced sense of control, self-awareness, and time control. The measurement was scored by using a 5-point Likert scale, ranging from 1 (never) to 5 (always), to rate the frequency of experienced flow. By summing the response values of the items, an overall flow experience tendency score is generated, ranging from 8 to 40. The higher the score, the higher the flow experience. SDFS-2 currently has a mature Chinese version and its reliability, validity, and stability are relatively good (Liu et al. 2012). Subsequent studies have further verified the effectiveness of the scale in measuring Chinese survey samples (Chen et al. 2022). Sample items are “I feel I am competent enough to meet the high demands of the situation” and “I am completely focused on the task at hand”.

#### 2.2.4. Creative Self-Efficacy

The dimension of creative self-efficacy for graduate students was assessed using the Short Scale of Creative Self (SSCS) developed by Carmeli and Schaubroeck (2007). The scale consists of eight items and participants rate their confidence in their creative abilities on a 5-point Likert scale, where 1 = absolutely not and 5 = absolutely yes. The scale has a mature Chinese version, of which the test has been supported by Chinese samples, with excellent reliability and structural validity (Han et al. 2022). Sample items are “I know I can efficiently solve even complicated problems” and “I trust my creative abilities”.

### 2.3. Data Analysis

In this study, SPSS 21 and AMOS 23 were used for data analysis. Firstly, descriptive statistics were conducted using SPSS to calculate the mean and standard deviation of each dimension in this study and the Pearson correlation test to analyze the correlation between graduate students’ mindfulness traits, scientific research creativity, flow experience, and creative self-efficacy. Secondly, a structural equation model was used to examine the path relationship between the four variables. Based on 5000 random repeated samples, the 95% confidence interval of the mediating effect was determined using the deviation-corrected nonparametric percentile Bootstrap method to test the chain mediating effect of flow experience and creative self-efficacy in the relationship between the mindfulness traits and scientific research creativity of graduate students. Finally, the effects of gender factors in the intermediary model were compared with a multi-group structural equation model.

Regarding conducting structural equation modeling, X^2^/Df, CFI, TLI, SRMR, and RMSEA are commonly used as fitting indicators that must be reported in general research. The following criteria are used to determine whether the data conform well to the hypothetical model: X^2^/Df is generally set to a standard of less than 10 and more stringent standards require less than 5; the qualification standard for CFI is 0.9; the TLI qualification standard is 0.9; SRMR should be less than 0.08; RMSEA should be less than 0.08 (Sharma et al. 2005; Schermelleh-Engel et al. 2003; Hu and Bentler 1999).

This study conducted a multi-group validated factor analysis (MGCFA) for gender subgroups, which was used to test the measurement invariance of the scale at the gender level. As shown in Table 1, we tested the weak-invariance model, strong-invariance model, and strict-invariance model. The *p*-values of ΔX2 for all three models were greater than 0.05 and ΔRMSEA and ΔCFI were less than 0.01. The results indicated support for the measurement invariance of the scale at the gender level.

## 3. Results

### 3.1. Common Method Variance Test

Harman’s one-way test was used to assess common method bias and exploratory factor analysis was conducted on all question items of the scale using SPSS 23 software. Using the criterion of eigenvalues greater than 1 before rotation, the first factor in the total variance explained with less than 40% of the explained variance (Hair et al. 1998) was considered not to have serious common method bias. The results showed that there were five factors with eigenvalues greater than one and the explained variance of the first factor in the total variance explained was 37%, which was less than the threshold of 40%, indicating that the data did not have a significant common method bias problem.

### 3.2. Descriptive Statistics and Reliability and Validity Tests

In this study, we used confirmatory factor analysis of the overall model, to assess the reliability and validity of each measurement indicator and the overall model. Regarding the mindfulness scale, Cronbach’s alpha coefficient of the scale is 0.879 and the relationship between each item and its respective latent variable is statistically significant; all indicators loading are 0.701–0.896 and square multiple correlations (SMC) are 0.558–0.911. and the convergent validity is 0.765. Regarding the scientific research creativity scale, Cronbach’s alpha coefficient of the scale is 0.950; all indicators loading are 0.763–0.976 and square multiple correlations (SMC) are 0.582–0.953. and the convergent validity is 0.837. Regarding the flow experience scale, Cronbach’s alpha coefficient of the scale is 0.902; all indicators loading are 0.778–0.893 and square multiple correlations (SMC) are 0.601–0.831. and the convergent validity is 0.815. Regarding the creative self-efficacy scale, Cronbach’s alpha coefficient of the scale is 0.955; all indicators loading are 0.733–0.910 and square multiple correlations (SMC) are 0.537–0.828. and the convergent validity is 0.723. Among them, convergent validity, also known as Average Variations Extracted (AVE), is derived from the formula AVE = (∑λ^2^)/n, where λ = factor loading and n = Number of indicators measured. All indicators of reliability and validity are good (Fornell and Larcker 1981; Hair et al. 1998).

Table 2 presents the descriptive statistics and correlation test results of the variables. Postgraduate students’ scientific research creativity was significantly and positively correlated with mindfulness traits (r = 0.468, *p* < 0.01). In addition, mindfulness traits were also significantly and positively correlated with flow experience (r = 0.664, *p* < 0.01) and creative self-efficacy (r = 0.814, *p* < 0.01). The correlation test results supported the follow-up analysis of structural equation models.

Table 2 also reports the sample distribution of the variables (skewness and kurtosis). Skewness is a measure of the degree of asymmetry or skewness in a distribution. Skewness is a dimensionless quantity that usually takes values ranging between −3 and +3, with larger absolute values indicating a greater degree of skewness. Kurtosis refers to the degree to which scores cluster around a central point. Kurtosis is zero in a normally distributed population (Lakes et al. 2012). The absolute values of kurtosis and skewness for all metrics in the table are less than 1, indicating that the overall sample distribution is unbiased (Kline 2023).

In addition, due to the high correlation between the variables, this study also assessed the discriminant validity. According to the average variance extracted (AVE) identification criterion proposed by Fornell and Larcker (1981), if the AVE square root value of each dimension (variable) is greater than the “maximum value of correlation coefficient of the dimension with other dimensions”, then it indicates good discriminant validity. As shown in Table 1 below, the square root of AVE for each dimension (variable) is greater than the correlation coefficient between that dimension and the other dimensions, which indicates that there is good discriminant validity among the variables of the measurement model.

### 3.3. Structural Equation Model Path Verification

**Figure 2:**
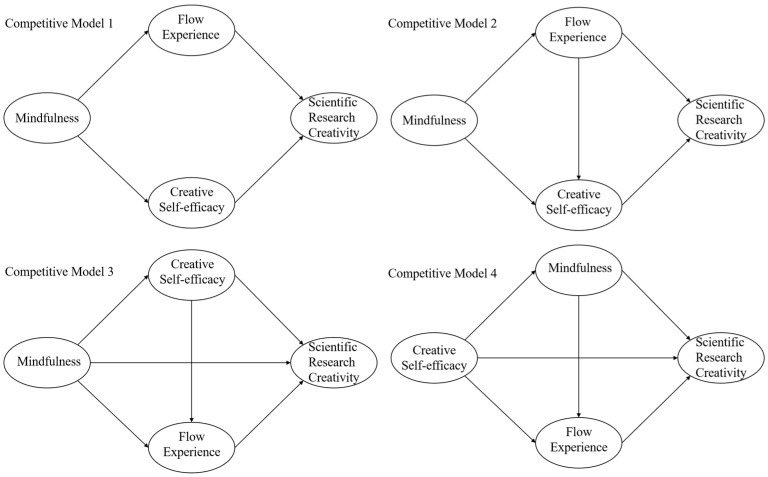
Competitive model.

In addition to constructing the hypothetical model in Figure 1, this study constructed four competing models, as shown in Figure 2. We supplemented with four competing models, namely, Competing Model 1 with fully mediated (without chain) mediation, Competing Model 2 with fully mediated (with chain) mediation, Competing Model 3 with a switched order of mediator variables, and Competing Model 4 with switched positions of mediator variables. Comparison between the competing model and the hypothetical model is usually considered for two reasons, one is that the goodness-of-fit index becomes better and the other is that the degree of explanation of the dependent variable is higher. The numerical comparison of the fit indexes of the competitive model and the hypothetical model, the comparison of the AIC and the R^2^ of the competitive model, are used to determine the appropriateness of the model selection. As shown in Table 3, it is found that the X^2^ values of Competitive Model 1 and Competitive Model 2 are significantly larger than the hypothetical model, the R^2^ value of Competitive Model 3 is smaller than the hypothetical model, and the AIC value of Competitive Model 4 is larger than the hypothetical model. Therefore, the data results are more supportive of the hypothetical model construction.

**Figure 3:**
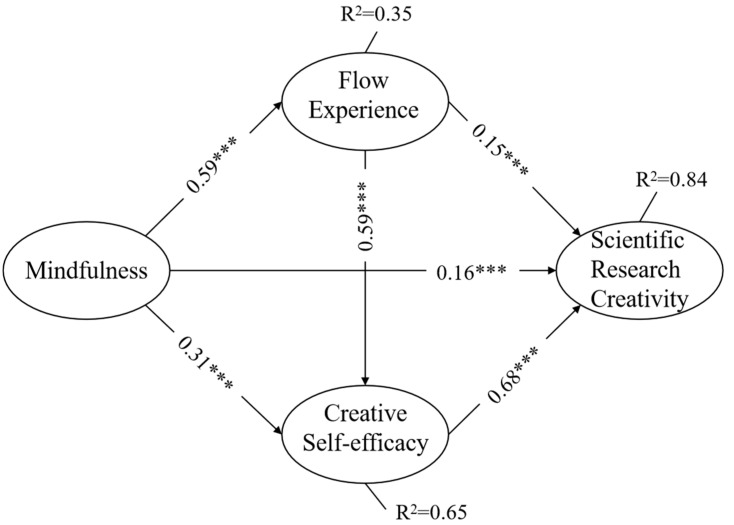
Structural equation model path result. *** *p* < 0.01.

The structural model is shown in Figure 3 and Table 4. In Table 2, the estimate is the non-standardized path coefficient. S.E. is to estimate the standard error of parameters and C.R. is the critical ratio; the critical ratio is the t-value of the t-test. If this value is greater than 1.96, it indicates a significance level of 0.05. The result of the model fitting index indicated that the data conformed well to the hypothetical model (X^2^ = 4015.44, df = 792, X^2^/df = 5.07, CFI = 0.91, TLI = 0.91, RMSEA = 0.07, SRMR = 0.05). The model path coefficient results suggested significant positive relationships between the mindfulness and flow experience of graduate students (β = 0.59, *p* < 0.001), as well as between mindfulness and creative self-efficacy (β = 0.31, *p* < 0.001). Additionally, flow experience (β = 0.15, *p* < 0.001) and creative self-efficacy (β = 0.68, *p* < 0.001) also showed a significant positive effect on graduate students’ scientific research creativity. Moreover, there was a significant positive influence of mindfulness on graduate students’ scientific research creativity (β = 0.16, *p* < 0.001). The R square value of flow experience is 0.35 and the R square value of creative self-efficacy is 0.65. The R square value of students’ scientific research creativity is 0.84, suggesting that the model as a whole is able to explain an 84% variance of students’ scientific research creativity.

The results are consistent with Hypothesis 1. Whether the mediating or chain mediating effect of flow experience and creative self-efficacy are tenable and the extent of the mediating effect require further research.

### 3.4. Mediation Effect Test

The mediating effects of flow experience and creative self-efficacy were tested by a bias-corrected nonparametric percentile Bootstrap method. The estimated mediating effect for each sampling was calculated to estimate the 95% confidence interval for the mediating effect (see Table 5). The results indicated that the estimated total effect point of mindfulness on graduate students’ scientific research creativity was 0.694 (Z > 1.96, 95% CI = [0.650, 0.737]) while the estimated total indirect effect point was 0.536 (Z > 1.96, 95% CI = [0.492, 0.583]). Through calculation, the total indirect effect proportion of mindfulness on graduate students’ scientific research creativity was 77.2%.

According to the effect of each intermediary variable, the estimated indirect effect point of individual flow experience was 0.091 (*p* < 0.01, 95% CI = [0.054, 0.135]), the estimated indirect effect point of individual creative self-efficacy was 0.208 (*p* < 0.01, 95% CI = [0.168, 0.253]), and the estimated chain indirect effect point from flow experience to creative self-efficacy was 0.237 (*p* < 0.01, 95% CI = [0.204, 0.275]). Comparing the mediating effects, it was found that the proportion of indirect effects of flow experience was 13.1%, the proportion of indirect effects of creative self-efficacy was 30.0%, and the proportion of indirect effects of chain mediation was 34.1%. To sum up, mindfulness played a role in graduate students’ scientific research creativity through the partial mediating effects of flow experience (13.1%), creative self-efficacy (30.0%), and chain mediation (34.1%). So, the assumption is that Hypothesis 2, Hypothesis 3, and Hypothesis 4 were valid.

### 3.5. Multi Group Model: Test on Gender Difference

**Figure 4:**
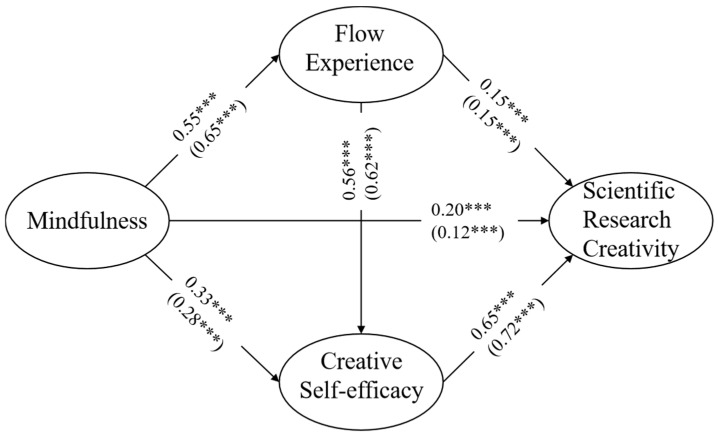
Multiple group structural equation model path results. *** *p* < 0.001. The figures are standardized regression coefficients, with coefficients for females outside the brackets and coefficients for males within the brackets.

To explore the differences in the influence of gender factors in the mediating model, we used a multi-group model to compare whether there were gender differences in the mediating effects of flow experience and creative self-efficacy. Based on constructing the male sample model and the female sample model, the path coefficients were analyzed (see Figure 4). The direct effect was lower in the males compared to the females (β _male_ = 0.12, *p* < 0.001; β _female_ = 0.20, *p* < 0.001) and the total mediating effect in males was higher than that in females (β _male_ = = 0.59, *p* < 0.001; β _female_ = 0.50, *p* < 0.001).

We also used the Z-test to determine whether there was a significant difference in the path coefficients between the male and female sample models and the results are shown in Table 6. In particular, there were significant differences between the male and female gender levels in the effects of mindfulness on flow experience and scientific research creativity and the effects of creative self-efficacy on research creativity (Z > 1.96, *p* < 0.05).

## 4. Discussion

This study explored the chain mediated effect of flow experience and creative self-efficacy on the relationship between mindfulness and graduate students’ scientific research creativity. The results suggested that mindfulness had a significant positive effect on graduate students’ scientific research creativity, which was mediated through three pathways: the single mediating effect of flow experience, the single mediating effect of creative self-efficacy, and the chain mediating effect of flow experience and creative self-efficacy.

### 4.1. The Effect of Mindfulness on Graduate Students’ Scientific Research Creativity

Mindfulness has a significant positive effect on graduate students’ scientific research creativity. The research results support previous research findings that mindfulness has a significant promoting effect on creativity (Gip et al. 2022; Ngo et al. 2020; Byrne and Thatchenkery 2019). Mindfulness can reduce habitual and unconscious behaviors in the learning process by maintaining flexible thinking and regulating emotions so as to play an important role in generating scientific research creativity.

Focusing on graduate students’ scientific research creativity is also one of the innovation points in this study, which enriches the relationship between mindfulness and research productivity. It is found that creativity can predict research productivity (Ramesh Babu and Singh 1998). Additionally, this study emphasizes the importance of mindfulness in fostering graduate students’ scientific research creativity, as well as the close relationship between mindfulness and research productivity. For graduate students, scientific research productivity, an important assessment standard, requires cognitive elaboration and insight into the processing of unstructured and unconventional tasks (Kim and Choi 2017). However, mindfulness promotes scientific research productivity by making graduate students focus and analyze things calmly, as well as break thinking patterns, engaging in divergent thinking (Bartlett et al. 2019). The above training can enable graduate students to focus on the present and expand their thinking in the level of self-awareness, emotional acceptance, and engagement in action, thereby improving their scientific research creativity.

### 4.2. Mediation of Flow Experience

The results revealed the important mediating role of flow experience in the relationship between mindfulness and scientific research creativity. We found that flow experience was influenced by mindfulness while mindfulness positively influenced flow experience, which also supports previous research findings (Wright et al. 2006; Chen et al. 2022). However, mindfulness might not always promote the flow experience. When the level of mindfulness exceeds a certain threshold, mindfulness can have an inhibitory effect on the flow experience. They have various development paths; for instance, mindfulness is required to maintain awareness of external stimuli and internal activities while flow experience needs a higher level of ecstasy to achieve complete immersion in a task (Sheldon et al. 2015).

Flow experience has a significant predictive effect on scientific research creativity, which verifies the view put forward by Csikszentmihalyi in 1997 that flow experience is an important prerequisite for high-level creativity (Csikszentmihalyi 1997). The generation of scientific research creativity requires the remote connection of thinking information and the reorganization of problem scenario information, which must be in a high degree of attention. Flow is recognized as a peak experience where the individual is highly immersed in tasks and this heightened state of engagement can lead to a notable increase in creativity (Cseh 2016).

### 4.3. Mediation of Creative Self-Efficacy

The relationship between mindfulness and graduate students’ scientific research creativity is partially mediated by creative self-efficacy. This finding is in line with previous research conducted by Mendonça et al. (2018), which also found that mindfulness positively predicts creative self-efficacy. When individuals focus their dispersed thoughts highly on the tasks at hand, they will pay more attention to related knowledge and information and then the integration and connection of a large amount of knowledge will generate new ideas (Dane and Brummel 2014). The rich knowledge reservation can help build confidence in generating new ideas and promote a sense of creative self-efficacy.

In addition, creative self-efficacy has a positive effect on scientific research creativity, suggesting that in the context of higher education, graduate students with a high sense of creative self-efficacy will be more persistent and diligent in facing challenging environments, believing in their ability to solve creative challenges, which has been also confirmed by similar conclusions (Chen and Zhang 2019; Liao et al. 2010). Some researchers have found that mentor academic support can improve graduate students’ creative self-efficacy (Gu et al. 2017). Therefore, mentors can improve graduate students’ self-confidence and flexibility by providing them with autonomy and emotional support and encouraging them to participate in creative activities, thereby enhancing their research creativity.

### 4.4. The Chain Mediating Effect of Flow Experience and Creative Self-Efficacy

Flow experience and creative self-efficacy play a chain mediating role between mindfulness and scientific research creativity. This study creatively uses flow experience and creative self-efficacy as chain mediating variables between mindfulness and scientific research creativity, with mindfulness accounting for approximately 77% of the indirect effect on scientific research creativity. It means that mindfulness has an effect on scientific research creativity through important mediating mechanisms. As an important pillar and powerful force of national development, graduate students should pay more attention to the training of logic and methods during their postgraduate studies due to their more difficult study, compared with undergraduate and high school students. Graduate students are required to complete challenging learning tasks, engage in divergent thinking, and come up with their own ideas (Luo et al. 2022). Thus, the important research topic is to explore the effect mechanism of graduate scientific research creativity, with mindfulness as the core independent variable so as to acknowledge the mechanism of mindfulness’s effect on research scientific creativity based on the chain mediating effect between mindfulness and graduate students’ scientific research creativity.

In addition, using a multi-group model, this study focused on gender differences in the effect of mindfulness on graduate students’ scientific research creativity. The results revealed that male students’ mindfulness had a lower direct effect on scientific research creativity than female students. This may be related to differences in gender physiological characteristics (Luders et al. 2015; Gong et al. 2022). Females exhibit more subtle emotions in their physiological characteristics that are relatively more likely to directly affect scientific research creativity through mindfulness.

### 4.5. Enhance Scientific Research Creativity to Cope with Anthropocene Challenges

The question of how to save nature and humanity itself through higher education in today’s Anthropocene has become an urgent educational question that needs to be answered. National education systems need to think about the fundamental future of education and, especially in this context, we have to think about how to prepare graduate students with the scientific creativity to deal with the new geological era of the Anthropocene and the pressing global issues that come with it (Barr et al. 2022). Fostering graduate students’ scientific research creativity through creative teaching in both formal and informal learning environments is essential if we are to successfully prepare graduate education for the uncertainties of the Anthropocene (Simonton 2012; Barr et al. 2022). Whereas our findings suggest that mindfulness can support the development of graduate student research creativity, mindfulness is a non-reactive awareness that enables one to live fully in the present moment (Maurits Kwee 2015). When students are mindful rather than distracted, they are able to think, innovate, and integrate, which, in turn, leads to sustainability-oriented innovations (Siqueira and Pitassi 2016). And they are able to address the enormous challenges associated with sustainability in the Anthropocene (Hensley 2020). Mindfulness is also a way to ameliorate fears, allow for uncertainty, and open up new creative possibilities—particularly by changing one’s relationship to uncertainty, becoming a better observer of the world, enabling greater openness to experience, and expanding empathy (Henriksen et al. 2022a).

Faculty members who purposefully incorporate positive thinking into the graduate student learning process through creative teaching and learning environments can benefit graduate students by fostering their scientific research creativity to cope with the uncertainties of the Anthropocene and the challenges of technological advancement (Henriksen et al. 2020). In addition, in this paper, we validate the mechanism of the influence of flow experience and creative self-efficacy as mediating variables, which is also designed to help educators understand how to better enhance graduate students’ scientific research creativity.

## 5. Conclusions and Limitations

This study aims to explore the relationship between mindfulness and graduate students’ scientific research creativity, especially for the chain mediating role of flow experience and creative self-efficacy. As we found, the relationship between graduate students’ mindfulness and scientific research creativity is positive. Meanwhile, flow experience and creative self-efficacy play a chain mediating role between mindfulness and graduate students’ scientific research creativity, as well as gender differences occurring in the direct relationship. The above results further reveal the mechanism of mindfulness on graduate students’ scientific research creativity, which is of great significance for improving graduate students’ scientific research creativity. It suggests that mindfulness meditation can improve graduate students’ scientific research creativity.

The limitations of this study include: (1) This study is a cross-sectional survey due to some limitations. Longitudinal follow-up studies can be conducted in the future to investigate long-term changes in variables and causal relationships; (2) The form of self-reporting in this study may not be as accurate as evaluations by others or some observable indicators. It may be considered to let graduate supervisors evaluate graduate students’ scientific research creativity in the future. As the first person in charge of graduate students’ academic output, supervisors can better understand the true level of graduate students’ scientific research creativity so it is necessary to collect questionnaires using a mentor–student pairing method or measure graduate students’ scientific research creativity with objective data, such as the quantity and quality of high-level papers.

## Figures and Tables

**Table 1:** Multi-group confirmatory factor analysis test for gender sub-groups.

Variable	ΔX^2^	Δdf	*p* Value	ΔRMSEA	ΔCFI
Metric/Weak-invariance model	24.123	24.000	0.455	0.001	0.001
Scalar/Strong-invariance model	41.231	37.000	0.291	0.001	0.001
Strict-invariance model	90.120	75.000	0.112	0.008	0.005

Note: Full metric invariance—with all factor loadings constrained equal. Scalar invariance—with all intercepts constrained equal. Strict invariance—with all factor loadings and intercepts fixed.

**Table 2:** Means, standard deviations, and correlations among variables in the research.

Variable	AVE	$\sqrt{\mathrm{AE}}V$	1	2	3	4
1. Mindfulness	0.693	0.832	—			
2. Flow Experience	0.694	0.833	0.664 **	—		
3. Creative Self-Efficacy	0.741	0.861	0.814 **	0.716 **	—	
4. Scientific Research Creativity	0.773	0.879	0.648 **	0.555 **	0.627 **	—
Mean	—	—	3.827	3.607	3.726	4.092
Standard deviation	—	—	0.539	0.707	0.745	0.765
Skewness	—	—	−0.295	0.272	0.086	−0.624
Kurtosis	—	—	0.863	0.262	−0.015	0.348

Note: ** *p* < 0.05.

**Table 3:** Comparison of multiple competing model fit metrics.

Variable	X^2^	df	X^2^/df	AIC	R^2^
Hypothetical Model	4015.44	792	5.07	4117.32	0.84
Competitive Model 1	4811.64	794	6.06	4982.76	0.74
Competitive Model 2	4470.22	794	5.63	4326.7.1	0.78
Competitive Model 3	4015.44	792	5.07	4117.32	0.82
Competitive Model 4	4015.44	792	5.07	4210.83	0.82

**Table 4:** Structural equation model path coefficients.

IV		DV	Estimate	S.E.	C.R.	*p*	Std
mindfulness	→	flow experience	0.646	0.028	22.806	***	0.594
mindfulness	→	creative self-efficacy	0.339	0.026	12.845	***	0.306
mindfulness	→	scientific research creativity	0.107	0.014	7.432	***	0.159
creative self-efficacy	→	scientific research creativity	0.412	0.021	19.258	***	0.68
flow experience	→	creative self-efficacy	0.596	0.026	22.900	***	0.585
flow experience	→	scientific research creativity	0.094	0.017	5.690	***	0.153

Note: Std means Standardized path coefficient, *** *p* < 0.01.

**Table 5:** Mediating results in the SEM.

Mediation Effect Test	Point Estimate	Bootstrap SE	Z	Bootstrapping 95% CI		*p*	Mediation Effect Proportion
				Lower	Upper		
Total effect	0.694	0.022	31.545	0.650	0.737	-	-
Direct effect	0.159	0.027	5.889	0.107	0.207	-	-
Indirect effect	0.536	0.021	25.524	0.492	0.583	0.007	77.2%
Indirect effect (MF→FE→SRC)	0.091	0.019	4.789	0.054	0.135	0.005	13.1%
Indirect effect (MF→CS→SRC)	0.208	0.022	9.455	0.168	0.253	0.007	30.0%
Indirect effect (MF→FE→CS→SRC)	0.237	0.017	13.941	0.204	0.275	0.120	34.1%

Note: SE means Standard Error, SRC means Scientific Research Creativity, FE means Flow Experience, CS means Creative Self-Efficacy, and MF means Mindfulness.

**Table 6:** Comparison of path coefficients in structural equation models of different genders.

IV		DV	Male		Female		Difference Z-Score Test
			Estimate	*p*	Estimate	*p*	
mindfulness	→	flow experience	0.722	<0.001	0.574	<0.001	−2.61 ***
mindfulness	→	creative self-efficacy	0.315	<0.001	0.355	<0.001	0.747
flow experience	→	creative self-efficacy	0.623	<0.001	0.575	<0.001	−0.914
mindfulness	→	scientific research creativity	0.094	<0.001	0.167	<0.001	1.992 **
creative self-efficacy	→	scientific research creativity	0.457	<0.001	0.371	<0.001	−2.029 **
flow experience	→	scientific research creativity	0.097	<0.001	0.086	<0.001	−0.312

Note: ** *p* < 0.05, *** *p* < 0.01, Estimate is an unstandardized regression coefficient.

## Data Availability

The data are currently not publicly available due to participant privacy; however, they are available from the corresponding author upon reasonable request.
